# Impact of Plasmatic Progesterone on the Day of Frozen Embryo Transfer in Hormone-induced Cycles

**DOI:** 10.1055/s-0041-1735229

**Published:** 2021-09-21

**Authors:** José Metello, Claudia Tomás, Pedro Ferreira, Iris Bravo, MaryJo Branquinho, Samuel Santos-Ribeiro

**Affiliations:** 1 Hospital Garcia de Orta, Almada, Portugal; 2 Instituto Valenciano de Infertilidad, Lisboa, Portugal

**Keywords:** progesterone, blastocyst, frozen embryo transfer, artificial cycle, miscarriage, delivery, progesterona, blastocisto, transferência de embrião congelado, ciclo artificial, aborto, parto

## Abstract

**Objective** To establish a relationship between serum progesterone values on the day of frozen blastocyst transfer in hormone-replaced cycles with the probability of pregnancy, miscarriage or delivery.

**Methods** This was an ambispective observational study including all frozen-thawed embryo transfer cycles performed at our department following in vitro fecundation from May 2018 to June 2019. The outcomes evaluated were β human chorionic gonadotropin (β-hCG)-positive pregnancy and delivery. Groups were compared according to the level of serum progesterone on the day of embryo transfer: the 1 ^st^ quartile of progesterone was compared against the other quartiles and then the 2 ^nd^ and 3 ^rd^ quartiles against the 4 ^th^ quartile.

**Results** A total of 140 transfers were included in the analysis: 87 with β-HCG > 10 IU/L (62%), of which 50 (36%) delivered and 37 had a miscarriage (42%). Women with lower progesterone levels (< 10.7ng/mL) had a trend toward higher β-HCG-positive (72 versus 59%; *p* > 0.05), lower delivery (26 versus 39%; *p* > 0.05) and higher miscarriage rates (64 versus 33%; *p* < 0.01). Comparing the middle quartiles (P25–50) with those above percentiles 75, the rate of pregnancy was similar (60 versus 57%; *p* > 0.05), although there was a trend toward a higher number of deliveries (43 versus 31%; *p* > 0.05) and a lower number of miscarriages (28 versus 45%; *p* > 0.05). These differences were not statistically significant.

**Conclusion** There were no differences in pregnancy and delivery rates related with the progesterone level when measured in the transfer day. The miscarriage rate was higher in the 1 ^st^ quartile group.

## Resumo

**Objetivo** Avaliar se existe alguma relação entre os valores plasmáticos de progesterona no dia da transferência de um blastocisto desvitrificado em ciclos hormonalmente substituídos e a taxa de gravidez, aborto ou nascido vivo.

**Métodos** Estudo observacional, ambispectivo, incluindo todos os ciclos de transferência de blastocistos congelados no nosso departamento, entre maio de 2018 e junho de 2019. Avaliou-se a taxa de gravidez e de nascidos vivos após 24 semanas de gestação. Os grupos foram comparados de acordo com os valores de progesterona plasmáticos dosados no dia da transferência do blastocisto: comparou-se o 1° quartil com os outros e depois os 2° e 3° quartis com o 4°.

**Resultados** Avaliaram-se 140 transferências: 87 com β gonadotrofina coriônica humana (β-HCG) > 10 IU/L (62%), 50 das quais terminaram em nascido vivo (36% do total), enquanto 37 tiveram um aborto (42% das gravidezes). Verificou-se uma tendência para menor número de recém-nascidos nas transferências com níveis de progesterona no 1° quartil (< 10.7ng/mL) (26 *versus* 39%; *p* > 0.05) e um maior número de abortos (64 *versus* 33%; *p* < 0.01). Comparando o 2° e 3° quartis com o 4°, verificou-se que nos casos em que a progesterona estava acima do percentil 75, apesar de uma taxa de gravidez semelhante (60 versus 57%; *p* > 0.05), houve uma tendência para uma maior taxa de nascidos vivos (43 versus 31%; *p* > 0.05) e menor número de abortos (28 versus 45%; *p* > 0.05) abaixo do percentil 75. Estas diferenças não foram estatisticamente significativas.

**Conclusão** Não se verificaram diferenças estatisticamente significativas para taxa de gravidez e de nascido vivo. A taxa de aborto foi maior no primeiro quartil.

## Introduction

Over the past decade, with the development of the vitrification technique and the consequent increase in embryo survival rate, frozen embryo transfers (FETs) have increased considerably. 1 The latest data published by the European Society of Human Reproduction and Embryology (ESHRE) shows that > 248,000 frozen embryo transfers or endometrial preparation cycles for oocyte donation embryos have been performed across Europe. 2 Some of the reasons behind this increase have been the use of elective embryo cryopreservation to reduce both the risk of hyperstimulation syndrome and the multiple pregnancy rate by following a single embryo transfer (SET) policy and, possibly, the intent of physicians to prevent a potential negative impact of supraphysiological hormonal levels during ovarian stimulation. 3 Moreover, another contributing factor is the increasing popularity of preimplantation genetic testing. 4

Endometrial preparation in FETs can be achieved in natural, modified, or artificial hormone substituted cycles, with no significant differences being reported to date in terms of clinical pregnancy and livebirth rates between the three preparation methods. 5 6 That said, the artificial hormone-substituted cycle has been most frequently used due to its ease in terms of scheduling and greater control of exogenous progesterone (P4) exposure, which is essential for achieving embryo-endometrial synchrony. 7 8

Endometrial receptivity appears to be related with the time and dose of P4 exposure after adequate estrogen exposure, since P4 is essential for embryo implantation and pregnancy outcome. 9 Exogenous P4 may be administered orally, vaginally, subcutaneously (SC) or intramuscularly (IM). Vaginal and IM administration were the most studied, with neither showing superiority over the other in terms of pregnancy outcomes. 10 However, the vaginal method is often preferred. 11 12 More recently, the oral route has also been evaluated as an alternative, showing to be non-inferior, at least when used in fresh embryo transfers. 12 13

Recent evidence suggests an important role of measuring serum progesterone values to predict the outcome of pregnancy. According to Gaggiotti-Marre et al., 14 a serum P4 value < 10.64 ng/mL on the day prior to FET of euploid embryos is associated with a higher miscarriage rate and with a lower live newborn rate. Another prospective observational study conducted on recipients of oocyte donor embryos found that a P4 value < 9.2 ng/mL on the day of FET is associated with a decreased rate of ongoing pregnancy. 15 On the other hand, although less consistently, high P4 levels (> 20 ng/mL) have also been associated with worse outcomes. 16

The aim of the present study was to evaluate if the serum P4 values on the day of frozen blastocyst transfer in hormone-replaced cycles are related with pregnancy, miscarriage, or delivery.

## Methods

This was an observational, ambispective study of FETs performed at the Centro de Infertilidade e Reprodução Medicamente Assistida (CIRMA) of the Hospital Garcia de Orta, Almada, Portugal, from May 2018 to June 2019, with the prospective collection of data commencing in September 2018.

All the cycles of women aged between 18 and 39 years old who had 1 or 2 frozen blastocysts transferred with an expansion degree ≥ 2 and with a grade 1 or 2 internal cell mass and trophectoderm (Istanbul Consensus, 2011) in a hormone-substituted cycle were included. 17

Patients with an endometrium < 6 mm prior to P4 administration, with endocavitary pathology or an uncorrected Mullerian anomaly, or those who obtained a P4 value not compatible with a luteal phase (< 2 ng/mL) were excluded from the analysis.

On the 2 ^nd^ day of a spontaneous or postpill menstrual cycle, the patient started estradiol (Zumenon®, Bayer Portugal, SA, Portugal) at a dose of 2mg vaginally each 12 hours. Ultrasound control was performed 12 to 20 days later. If the endometrial lining was trilaminar and with a thickness > 7 mm, the patients started vaginal administration of P4 (Progeff®, Laboratórios Effik, Portugal) at a dose of 400 mg each 12 hours starting on the following morning.

A serum P4 assay was performed on the day of ultrasound for confirmation of anovulation, and another one on the morning of the transfer after the 11 ^th^ vaginal P4 administration.

A maximum of 2 embryos were warmed according to the following protocol: the Cryotop straw (Kitazato, Japan) was removed from liquid nitrogen and immediately submerged in 300µl of thawing solution (Kitazato, Japan) previously heated to 37°C. After 1 minute, the embryos were placed in a 60µl drop of diluent solution (Kitazato, Japan) for 3 minutes at room temperature. Finally, they were placed in a 60µl drop of washing solution (Kitazato, Japan) for 5 minutes at room temperature, and then were washed for 1 minute in another drop of 60 µl of washing solution at room temperature. They were then placed into 30 µl drops of Sequential Blast medium (ORIGIO, Denmark) covered with Liquid Paraffin (ORIGIO, Denmark), where they remained for at least 2 hours prior to transfer.

Embryo transfers are routinely performed under ultrasound guidance in our center using either a Cook or Wallace embryo catheter introduced until it passes the middle of the endometrial cavity, where the embryos are deposited. All transfers were performed by physicians with at least 100 previously performed transfers. One or two blastocysts were transferred. The β-HCG test was performed 9 to 12 days after the transfer and, in those who conceived, estradiol and P4 were maintained until the 12 ^th^ week of pregnancy.

Progesterone and β-HCG hormone assays were performed using electrochemiluminescence (ECLIA) and the Modular EVO E170 Roche Diagnostics (Roche Holding AG, Basel, Switzerland) equipment. The β-HCG assay method was based on sandwich-type immunological reaction, and the P4 assay on competitive immunological reaction.

A β-HCG-positive pregnancy was considered in all cycles with a serum β-HCG value > 10 IU/L. Delivery was considered in all pregnancies delivering a liveborn after 24 weeks. The miscarriage rate was calculated as the difference between delivery rate and a β-HCG value > 10 IU/L.

We estimated a delivery rate of 50% in the highest quartile of patients against 25% in lowest one. In this case, considering an α-error of 5% with an 80% power, we estimated that 232 cases were needed for analysis.

The following variables were subject to statistical analysis: female age on the day of the oocyte retrieval, body mass index (BMI) of women and men, smoking habits of women and men, ethnicity of women and men, anti-Mullerian hormone (AMH) value, antral follicle count (AFC), total dose of gonadotropins used in the IVF/ICSI cycle, number of oocytes and embryos obtained, blastocyst development day (D5 or D6), number of transferred embryos, FET rank, endometrium thickness prior to FET, serum P4 value on the day of FET, and transfer difficulty rating (easy or difficult).

A percentile distribution was made according to the serum P4 levels measured on the day of FET.

A p-value < 0.05 was considered statistically significant. Continuous variables were compared with the Mann-Whitney test, and discrete variables with the chi-squared test. IBM SPSS Statistics for Windows, version 22.0 (IBM Corp., Armonk, NY, USA) software was used.

The present study was approved by the Ethics Committee of the Hospital Garcia de Orta. Informed consent was obtained from patients followed prospectively ( *n* = 102) on the day of the FET scheduling consultation.

## Results

**Fig. 1:**
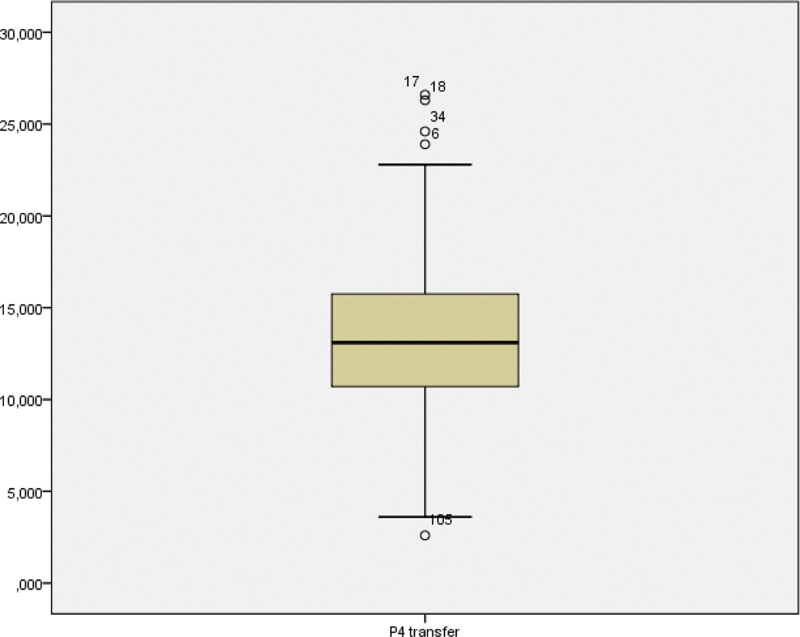
Progesterone steam and leaf distribution of progesterone (ng/mL).

The investigation team decided to prematurely terminate the study for ethical reasons after the publication of several studies in 2019 suggesting worse results in women with lower P4 values. A review of the 140 cases already included in the study at the time was performed, with 38 being retrospective and 102 prospective. There were 87 cases with β-HCG > 10 IU/L (62%), 50 cases with delivery (36%), and 37 cases of miscarriage (42%). The P4 concentration on the day of the embryo transfer varied between 2.6 and 26.6 ng/mL ( Fig. 1 ). Women were grouped in 4 quartiles according to these values. The 1 ^st^ quartile (P4 < 10.7 ng/mL) was compared with the rest. As shown in Table 1 , women with lower P4 levels had a higher number of pregnancies (71 versus 59%) but a lower rate of deliveries (26 versus 39%), although these differences were not statistically significant. Moreover, the miscarriage rate was almost double among women with lower P4 levels (64 versus 33%; *p* < 0.01).

**Table 1:** Comparison between p25, p25–100

	Progesterone < 10.7 ng/mL ( *n* = 35)	Progesterone > 10.7 ng/mL ( *n* = 105)	*p-value*
β-HCG +	71%	59%	0.191
Deliveries	26%	39%	0.154
Miscarriage	64%	33%	0.009

All the other variables evaluated were compared between groups ( Tables 2 and 3 ). Although no statistically significant differences were found, in the group with lower P4 values, there were more day 6 blastocyst transfers (31 versus 18%) and more transfers ranked as being the 1 ^st^ (77 versus 69%). The only statistically significant difference was male BMI (28.3 versus 24.9).

**Table 2:** Comparison between p25, p25–100

	Progesterone <10.7 ng/mL ( *n* = 35)	Progesterone > 10.7 ng/mL ( *n* = 105)	*p* - *value*
**Female smoking habits** *n (%)*			
Before	7 (20%)	16 (15%)	0.793
Never	20 (57%)	62 (59%)	
Present	8 (23%)	27 (26%)	
Male smoking habits *n (%)*			
Before	7 (20%)	16 (15%)	0.368
Never	18 (51%)	45 (43%)	
Present	10 (29%)	44 (42%)	
Female ethnicity *n (%)*			
Caucasian	29 (83%)	93 (89%)	0.382
Noncaucasian	6 (17%)	12 (11%)	
Male ethnicity *n (%)*			
Caucasian	31 (89%)	92 (88%)	0.881
Noncaucasian	4 (11%)	13 (12%)	
Infertility cause *n (%)*			
Idiopatic	11 (31%)	23 (22%)	0.517
Endometriosis	6 (17%)	18 (17%)	
Ovulatory	1 (3%)	13 (12%)	
Tubar	1 (3%)	12 (11%)	
Multiple female	1 (3%)	2 (2%)	
Male factor	11 (31%)	28 (27%)	
Male and female factors	4 (11%)	7 (7%)	
Other	0 (0%)	2 (2%)	
Fecundation method *n (%)*			
IVF	22 (63%)	65 (62%)	0.965
ICSI	12 (34%)	36 (34%)	
Mixed	1 (3%)	4 (4%)	
Embryo development *n (%)*			
D5 blastocyst	24 (69%)	86 (82%)	0.096
D6 blastocyst	11 (31%)	19 (18%)	
Number of transferred embryos *n (%)*			
1 embryo	28 (80%)	82 (78%)	0.812
2 embryos	7 (20%)	23 (22%)	
FET rank *n (%)*			
First	27 (77%)	72 (69%)	0.505
Second	7 (20%)	25 (24%)	
Third	1 (3%)	8 (8%)	

Abbreviations: FET, Frozen embryo transfer; IVF, In vitro Fertilization; ICSI, Intracytoplasmic injection.

**Table 3:** Comparison between p25, p25–100

	Progesterone < 10.7 ng/mL ( *n* = 35)	Progesterone > 10.7 ng/mL ( *n* = 105)	*p* - *value*
Female age (mean/SD)	34.3 (3.53)	34.1 (3.79)	0.813
Duration of infertility (months) (mean/SD)	62.2 (27.8)	59.3 (26.1)	0.346
Endometrial thickness (mm) (mean/SD)	9.65 (1.99)	9,47 (1.86)	0.791
Female weight (kg) (mean/SD)	67.3 (14.1)	66.2 (15.0)	0.640
Female BMI (kg/m ^2^ ) (mean/SD)	25.3 (5.1)	24,96 (5.2)	0.657
Male age (mean/SD)	36.3 (5.5)	35.1 (4.8)	0.372
Male BMI (kg/m ^2^ ) (mean/SD)	28.3 (5.4)	24.9 (3.6)	**0.000**
AMH (ng/mL) (mean/SD)	3.54 (3.7)	3.84 (3.3)	0.292
AFC (mean/SD)	18.2 (12.5)	19.8 (12.4)	0.398
Total gonadotropin dose (UI) (mean/SD)	2863 (805)	2738 (770)	0.367
Number of oocytes retrieved (mean/SD)	14.0 (9.0)	15.1 (7.4)	0.305
Number of MII oocytes (mean/SD)	13.4 (8.8)	13.9 (7.1)	0.399
Number of obtained embryos (mean/SD)	9.7 (6.5)	9.2 (6.1)	0.921
Number of cryopreserved embryos (mean/SD)	3.5 (2.5)	3.4 (2.3)	0.868

Abbreviations: AFC, Antral follicle count; AMH, Anti-mullerian hormone; BMI, Body mass index; MII, metaphase II; SD, standard deviation.

A comparison was also made between percentiles 25-75 (P4 10.7 to 15.7 ng/mL) and > 75 (P4 > 15.7 ng/mL). The results are shown in Table 4 . The rate of pregnancy was similar (60 versus 57%), although there was a trend toward a higher number of deliveries (43 versus 31%) and a lower number of miscarriages (28 versus 45%) in the P4 25-75 percentile group. However, this difference was not statistically significant.

**Table 4:** Comparison between p 25–75 and over p75

	Progesterone 10.7–15.7 ng/mL ( *n* = 70)	Progesterone > 15.7 ng/mL ( *n* = 35)	*p-value*
β-HCG +	60%	57%	0.779
Deliveries	43%	31%	0.258
Miscarriage	28%	45%	0.180

### Progesterone Variation among Women

Given the trend toward worse outcomes in the extremes, a correlation between age, weight, height and BMI was performed in relation to progesterone values. There is not any statistically significant correlation between P4 values and weight or BMI.

## Discussion

The present study was designed to evaluate whether P4 values measured on the transfer day of a blastocyst in a FET cycle were related with pregnancy, delivery, or miscarriage rates. Unfortunately, the study was terminated prematurely due to ethical concerns related to the loss of study equipoise. The evaluation of the results showed a trend toward a worse outcome amongst women with lower P4 levels and in those with higher values (delivery rate for P4 < 10.7 ng/mL: 26% versus 43% for P4 between 10.7 and 15.7 ng/mL versus 31.4% when P4 is over 15.7ng/mL). Currently, there are several studies published regarding the existence of a minimal cutoff of serum P4 value to be achieved in hormone-substituted FET cycles, under which there appears to be a significant decrease in pregnancy rates ( Table 5 ).

**Table 5:** Summary of published articles

Author, journal, year	Country	Type of study	*n*	Oocytes origin	Number of D3/D5 embryos transferred	P4 administration route and dosage	Better cutoff	Day of the analysis	OPR / LBR *
Brady et al. (2014) 18 J Assist Reprod Genet	USA	Retrospective	229	donors	1 to 3 embryos in D3	IM 50–100 mg	> 20 ng/mL (64 nmol/l)	4 ^th^ day of administration = FET day	65 VS 51%
Kofinas et al. (2015) 16 J Assis Repro Gen	USA	Retrospective	213	autologous	1 euploid blastocysts	Progesterone IM 50–75 mg	< 20 ng/mL	2 ^nd^ and e 6 ^th^ days of administration	49 VS 65%
Yovich et al. (2015) 19 RBM Online	Australia	Retrospective	529	autologous and donors	1 vitrified blastocyst	vaginal 400mg 3id **	50–100 nmol/L	8 ^th^ /9 ^th^ day of administration	50% VS 41%/36% ***
Labarta et al. (2017) 15 Human Reprod	Spain	Prospective	211	donors	vitrified blastocysts	vaginal 400 mg 2id	> 11 ng/mL (> 35 nmol/L)	6 ^th^ day of administration = FET day	33 VS 53%
Basnayake et al. (2018) 20 Aust N Z J Obstet Gynaecol	Australia	Retrospective	1580 ▪	autologous and donors	vitrified clivage and blastocysts □	vaginal in different doses	> 50 nmol/L (15,7 ng/mL)	16 ^th^ day of administration	26 VS 11%
Alsbjerg et al. (2018) 21 RBM Online	Denmark	Retrospective	244	autologous	1 or 2 vitrified blastocysts	vaginal 90 mg 3id	> 35 nmol/L	9 ^th^ –11 ^th^ day of administration = β-HCG day	31% vs 51%
Gaggiotti-Marre et al. (2018) 14 Gynecological Endocr	Spain	Retrospective	244	autologous	euploid vitrified blastocysts	vaginal 200 mg 3id	10.64 ng/mL	4 ^th^ day of admnistration	59.6 VS 41%
Cédrin-Durnerin et al. (2018) 22 RBM Online	France	Retrospective	227	??	vitrified clivage and blastocysts	vaginal 200 mg 3id	10.7–12.3 ng/mL	6 ^th^ day of administration = FET day	31 VS 17%
Labarta et al. (2019) 23 data from ESHRE congress abstract – Hum Reprod	Spain	Prospective	1155	autologous and donors	vitrified blastocysts	vaginal 400 mg 2id	> 8.8 ng/mL	6 ^th^ day of administration = FET day	58 VS 40%

Abbreviations: FET, Frozen embryo transfer; LBR, Live-birth rates; OPR, Ongoing pregnancy rates; P4, Progesterone.

* above and below cutoff levels, respectively.

** pessaries produced for themselves.

*** 50% corresponding to the interval rate VS above/below cutoff levels.

▪ multicentric.

□ slow freezing and vitrification.

Generally, higher P4 values are often associated with better outcomes in FETs. However, some studies suggest a maximum serum P4 value above which a decrease in pregnancy rates and an increase in miscarriage rates may become evident. 16 19

Except for the studies by Labarta et al., 15 23 all studies are retrospective and, therefore, have the biases inherent to this type of study. Moreover, they are highly variable regarding the type and dose of P4 administered on the day of dosing as well as the knowledge of embryo ploidy and their cleaved or blastocyst status, thus reflecting the heterogeneity of clinical practice.

In natural cycles, the adequate value of produced P4 by the corpus luteum seems to be ∼ 10 ng/mL, 24 25 although their values may fluctuate from cycle to cycle. 26 According to the endometrial implantation window theory, 7 there is an optimal period for embryonic implantation, which is between the 19 ^th^ and 20 ^th^ day and may last up to 4 or 5 days; in other words, 5 to 9 days after ovulation, midway through the luteal and secretory phase. 27 However, its duration may vary inter- and intracyclically in each menstrual cycle. 7 Therefore, based on this implantation window and considering the minimum and maximum cutoff of serum P4, transferring an embryo outside these values may correspond to an asynchrony between endometrial and embryonic development, reducing implantation rates. 16 18 21 Low P4 may delay the implantation window, while higher P4 may advance the implantation window, thus shifting the transfer from the optimal period for embryo implantation. For the first case, one may speculate that extra P4 supplementation may be considered; on the other hand, in the second case, a reduction in the dose could be an option.

In the case of low P4 values, an effective “rescue” strategy is yet to be established. In the studies by Cédrin-Durnerin et al. 22 with vaginal P4 and by Brady et al. 18 with IM P4, on the day of P4 dosing (6 ^th^ and 4 ^th^ day of therapy, both corresponding to the day of FET), if the serum P4 values were < 10 and < 20 ng/mL, respectively, the supplementation administered to the patient would be doubled in the first study (400 mg 3id) and, in the second study, the IM P4 dose would be increased by between 50 and 100%. 22 In both cases, a new dosing was performed 2 to 4 days after the P4 dose increase. Despite supplementation, these groups maintained worse rates of ongoing pregnancy and livebirth rates, which may demonstrate that the supplementation adjustments were ineffective, possibly because the correction might have been made too late. In this case, the ideal may be to dose P4 on the 2 ^nd^ or 3 ^rd^ day of administration, soon after reaching its steady state, to rescue the cycle in a timely manner.

Although proximate, the different values of minimal serum P4 found suggest that the absorption and metabolization in each patient is very variable, warranting monitoring during FET. 15 18 28 There are few studies evaluating the variation in P4 levels among women on the same dosage and administration interval. The vaginal method directly reaches the uterus (uterine first pass effect), avoiding the hepatic first pass effect and its inherent metabolism, leading to serum levels that are higher and more sustained than the oral route and to higher endometrial levels than the IM or SC routes. 28 29 30 Therefore, results obtained for serum values with one specific method of administration should not be extrapolated to another method.

As strengths of the present study, we highlight the fact that most of the data ( *n* = 102) was collected prospectively in a single center and in a relatively short period. Moreover, only autologous oocytes were used, with only blastocysts being transferred and with the same route of administration and measurement of P4.

As potential limitations, we must mention the number of cases, which, although suggesting worse outcome in the extremes, was insufficient to safely determine cutoff values that can predict worst results. Moreover, patients with a transfer of either one or two embryos were included, which, despite being of good quality, contributed to the heterogeneity of our sample. Another limitation is the fact that none of the embryos was genetically screened before transfer, which means that, by random effect, aneuploidy embryos could have been more frequent in the 1 ^st^ quartile and, in fact, be responsible for such a higher miscarriage rate.

## Conclusion

The measurement of serum P4 on the day of transfer of frozen embryos can be important to improve results. Women with lower P4 levels (< 10.7 ng/mL) had more miscarriages. No statistically significant results were identified in women with lower (< 10.7 ng/mL) or higher (15.7 ng/mL) P4 compared with intermediate ones.

## References

[1] Rienzi L Gracia C Maggiulli R LaBarbera A R Kaser D J Ubaldi F M Oocyte, embryo and blastocyst cryopreservation in ART: systematic review and meta-analysis comparing slow-freezing versus vitrification to produce evidence for the development of global guidance Hum Reprod Update 2017 23 02 139 155 10.1093/humupd/dmw038 27827818 PMC5850862

[2] European IVF-monitoring Consortium (EIM) for the European Society of Human Reproduction and Embryology (ESHRE) De Geyter C Calhaz-Jorge C Kupka M S Wyns C Mocanu E Motrenko T ART in Europe, 2014: results generated from European registries by ESHRE: The European IVF-monitoring Consortium (EIM) for the European Society of Human Reproduction and Embryology (ESHRE) Hum Reprod 2018 33 09 1586 1601 10.1093/humrep/dey242 30032255

[3] Devroey P Polyzos N P Blockeel C An OHSS-Free Clinic by segmentation of IVF treatment Hum Reprod 2011 26 10 2593 2597 10.1093/humrep/der251 21828116

[4] Coates A Kung A Mounts E Hesla J Bankowski B Barbieri E Optimal euploid embryo transfer strategy, fresh versus frozen, after preimplantation genetic screening with next generation sequencing: a randomized controlled trial Fertil Steril 2017 107 03 723 730000 10.1016/j.fertnstert.2016.12.022 28139240

[5] Groenewoud E R Cohlen B J Macklon N S Programming the endometrium for deferred transfer of cryopreserved embryos: hormone replacement versus modified natural cycles Fertil Steril 2018 109 05 768 774 10.1016/j.fertnstert.2018.02.135 29778369

[6] Yarali H Polat M Mumusoglu S Yarali I Bozdag G Preparation of endometrium for frozen embryo replacement cycles: a systematic review and meta-analysis J Assist Reprod Genet 2016 33 10 1287 1304 10.1007/s10815-016-0787-0 27549760 PMC5065562

[7] Blesa D Ruiz-Alonso M Simón C Clinical management of endometrial receptivity Semin Reprod Med 2014 32 05 410 413 10.1055/s-0034-1376360 24959823

[8] El-Toukhy T Coomarasamy A Khairy M Sunkara K Seed P Khalaf Y The relationship between endometrial thickness and outcome of medicated frozen embryo replacement cycles Fertil Steril 2008 89 04 832 839 10.1016/j.fertnstert.2007.04.031 17681313

[9] Gellersen B Brosens J J Cyclic decidualization of the human endometrium in reproductive health and failure Endocr Rev 2014 35 06 851 905 10.1210/er.2014-1045 25141152

[10] van der Linden M Buckingham K Farquhar C Kremer J A Metwally M Luteal phase support for assisted reproduction cycles Cochrane Database Syst Rev 2015 07 CD009154 10.1002/14651858.CD009154.pub3 26148507 PMC6461197

[11] Casper R F Yanushpolsky E H Optimal endometrial preparation for frozen embryo transfer cycles: window of implantation and progesterone support Fertil Steril 2016 105 04 867 872 10.1016/j.fertnstert.2016.01.006 26820769

[12] Griesinger G Tournaye H Macklon N Petraglia F Arck P Blockeel C Dydrogesterone: pharmacological profile and mechanism of action as luteal phase support in assisted reproduction Reprod Biomed Online 2019 38 02 249 259 10.1016/j.rbmo.2018.11.017 30595525

[13] Rashidi B H Ghazizadeh M Tehrani Nejad E S Bagheri M Gorginzadeh M Oral dydrogesterone for luteal support in frozen-thawed embryo transfer artificial cycles: a pilot randomized controlled trial Asian Pac J Reprod 2016 5 06 490 494 10.1016/j.apjr.2016.10.002

[14] Gaggiotti-Marre S Martinez F Coll L Garcia S Álvarez M Parriego M Low serum progesterone the day prior to frozen embryo transfer of euploid embryos is associated with significant reduction in live birth rates Gynecol Endocrinol 2019 35 05 439 442 10.1080/09513590.2018.1534952 30585507

[15] Labarta E Mariani G Holtmann N Celada P Remohí J Bosch E Low serum progesterone on the day of embryo transfer is associated with a diminished ongoing pregnancy rate in oocyte donation cycles after artificial endometrial preparation: a prospective study Hum Reprod 2017 32 12 2437 2442 10.1093/humrep/dex316 29040638

[16] Kofinas J D Blakemore J McCulloh D H Grifo J Serum progesterone levels greater than 20 ng/dl on day of embryo transfer are associated with lower live birth and higher pregnancy loss rates J Assist Reprod Genet 2015 32 09 1395 1399 10.1007/s10815-015-0546-7 26238390 PMC4595397

[17] ALPHA Scientists In Reproductive Medicine ESHRE Special Interest Group Embryology Istanbul consensus workshop on embryo assessment: proceedings of an expert meeting Reprod Biomed Online 2011 22 06 632 646 10.1016/j.rbmo.2011.02.001 21481639

[18] Brady P C Kaser D J Ginsburg E S Ashby R K Missmer S A Correia K F Serum progesterone concentration on day of embryo transfer in donor oocyte cycles J Assist Reprod Genet 2014 31 05 569 575 10.1007/s10815-014-0199-y 24619510 PMC4016380

[19] Yovich J L Conceicao J L Stanger J D Hinchliffe P M Keane K N Mid-luteal serum progesterone concentrations govern implantation rates for cryopreserved embryo transfers conducted under hormone replacement Reprod Biomed Online 2015 31 02 180 191 10.1016/j.rbmo.2015.05.005 26099447

[20] Basnayake S K Volovsky M Rombauts L Osianlis T Vollenhoven B Healey M Progesterone concentrations and dosage with frozen embryo transfers - What's best? Aust N Z J Obstet Gynaecol 2018 58 05 533 538 10.1111/ajo.12757 29271471

[21] Alsbjerg B Thomsen L Elbaek H O Laursen R Povlsen B B Haahr T Progesterone levels on pregnancy test day after hormone replacement therapy-cryopreserved embryo transfer cycles and related reproductive outcomes Reprod Biomed Online 2018 37 05 641 647 10.1016/j.rbmo.2018.08.022 30385142

[22] Cédrin-Durnerin I Isnard T Mahdjoub S Sonigo C Seroka A Comtet M Serum progesterone concentration and live birth rate in frozen-thawed embryo transfers with hormonally prepared endometrium Reprod Biomed Online 2019 38 03 472 480 10.1016/j.rbmo.2018.11.026 30642638

[23] Labarta E D Mariani G Paolelli S O-173: A large prospective trial in unselected population confirms that low serum progesterone on the day of embryo transfer impairs pregnancy outcome in artificial cycles. Hum Reprod 2019;34 Suppl 1:82. Abstract Book - ESHRE 2019–Vienna, Austria, 2019 Jun 23–26.

[24] Hull M G Savage P E Bromham D R Ismail A A Morris A F The value of a single serum progesterone measurement in the midluteal phase as a criterion of a potentially fertile cycle (“ovulation”) derived form treated and untreated conception cycles Fertil Steril 1982 37 03 355 360 10.1016/s0015-0282(16)46095-4 7060786

[25] Jordan J Craig K Clifton D K Soules M R Luteal phase defect: the sensitivity and specificity of diagnostic methods in common clinical use Fertil Steril 1994 62 01 54 62 10.1016/s0015-0282(16)56815-0 8005304

[26] Murray M J Meyer W R Zaino R J Lessey B A Novotny D B Ireland K A critical analysis of the accuracy, reproducibility, and clinical utility of histologic endometrial dating in fertile women Fertil Steril 2004 81 05 1333 1343 10.1016/j.fertnstert.2003.11.030 15136099

[27] Lessey B A Assessment of endometrial receptivity Fertil Steril 2011 96 03 522 529 10.1016/j.fertnstert.2011.07.1095 21880273

[28] Nahoul K Dehennin L Jondet M Roger M Profiles of plasma estrogens, progesterone and their metabolites after oral or vaginal administration of estradiol or progesterone Maturitas 1993 16 03 185 202 10.1016/0378-5122(93)90064-o 8515718

[29] Miles R A Paulson R J Lobo R A Press M F Dahmoush L Sauer M V Pharmacokinetics and endometrial tissue levels of progesterone after administration by intramuscular and vaginal routes: a comparative study Fertil Steril 1994 62 03 485 490 10.1016/s0015-0282(16)56935-0 8062942

[30] Paulson R J Collins M G Yankov V I Progesterone pharmacokinetics and pharmacodynamics with 3 dosages and 2 regimens of an effervescent micronized progesterone vaginal insert J Clin Endocrinol Metab 2014 99 11 4241 4249 10.1210/jc.2013-3937 24606090

