# Corrigendum

**DOI:** 10.1055/s-0041-1736410

**Published:** 2021-10-04

## Corrigendum

São Paulo, September 22, 2021

Dear readers,

In the Article *Impact of Plasmatic Progesterone on the Day of Frozen Embryo Transfer in Hormone-induced Cycles* ( *Rev Bras Ginecol Obstet* . 10.1055/s-0041-1735229 .), published online in *Rev Bras Ginecol Obstet* in September 2021.

**Where it reads:**

José Metello1 Claudia Tomás1 Iris Bravo1 MaryJo Branquinho1 Samuel Santos-Ribeiro2

**It should read:**

José Metello1 Claudia Tomás1 Pedro Ferreira1 Iris Bravo1 MaryJo Branquinho1 Samuel Santos-Ribeiro2

